# Dissemination and persistence of extended-spectrum cephalosporin-resistance encoding IncI1-*bla*_CTXM-1_ plasmid among *Escherichia coli* in pigs

**DOI:** 10.1038/s41396-018-0200-3

**Published:** 2018-06-13

**Authors:** Sam Abraham, Roy N. Kirkwood, Tanya Laird, Sugiyono Saputra, Tahlia Mitchell, Mohinder Singh, Benjamin Linn, Rebecca J. Abraham, Stanley Pang, David M. Gordon, Darren J. Trott, Mark O’Dea

**Affiliations:** 1 Antimicrobial Resistance and Infectious Diseases Laboratory, School of Veterinary and Life Sciences, Murdoch University, Melbourne, Western Australia Australia; 2 School of Animal and Veterinary Sciences, University of Adelaide, Roseworthy, Australia; 3 Australian Centre for Antimicrobial Resistance Ecology, University of Adelaide, Adelaide, Australia; 4 Research Center for Biology, Indonesian Institute of Sciences, West Java, Cibinong, Indonesia; 5 Evolution, Ecology and Genetics, Research School of Biology, The Australian National University, Canberra, Australia

## Abstract

This study investigated the ecology, epidemiology and plasmid characteristics of extended-spectrum cephalosporin (ESC)-resistant *E. coli* in healthy pigs over a period of 4 years (2013–2016) following the withdrawal of ESCs. High carriage rates of ESC-resistant *E. coli* were demonstrated in 2013 (86.6%) and 2014 (83.3%), compared to 2015 (22%) and 2016 (8.5%). ESC resistance identified among *E. coli* isolates was attributed to the carriage of an IncI1 ST-3 plasmid (pCTXM1-MU2) encoding *bla*_CTXM-1_. Genomic characterisation of selected *E. coli* isolates (*n* = 61) identified plasmid movement into multiple commensal *E. coli* (*n* = 22 STs). Major STs included ST10, ST5440, ST453, ST2514 and ST23. A subset of the isolates belong to the atypical enteropathogenic *E. coli* (aEPEC) pathotype that harboured multiple LEE pathogenic islands. pCTXM1-MU2 was similar (99% nt identity) to IncI1-ST3 plasmids reported from Europe, encoded resistance to aminoglycosides, sulphonamides and trimethoprim, and carried colicin *Ib*. pCTXM1-MU2 appears to be highly stable and readily transferable. This study demonstrates that ESC resistance may persist for a protracted period following removal of direct selection pressure, resulting in the emergence of ESC-resistance in both commensal *E. coli* and aEPEC isolates of potential significance to human and animal health.

## Introduction

The World Health Organisation (WHO) has recently warned of a post-antibiotic era after discovering widespread occurrence of antimicrobial resistance (AMR) to critically important antimicrobials (CIAs), including extended-spectrum cephalosporins (ESC), fluoroquinolones (FQ), carbapenems and colistin in bacteria that cause serious infections in humans worldwide [1]. There are also concerns of CIA-resistant bacteria originating from food-producing animals through direct transfer of zoonotic pathogens such as multidrug-resistant (MDR) *Salmonella* spp. and *Escherichia coli* as well as indirect transfer of mobile AMR genes to commensal or pathogenic human bacteria through the food chain [2, 3].

Globally, there is much debate concerning antimicrobial usage in food-producing animals [4]. The development of AMR is complex, driven by both clonal expansion of MDR strains and horizontal transmission of mobile genetic elements carrying AMR genes, including plasmids, transposons and integrons, under antibiotic selection pressure [3]. In intensive livestock production systems, the emergence of CIA-resistance among commensal *E. coli* may lead to an increased resistance burden in the gut microbiota [5, 6].

Ceftiofur and cefquinome are ESCs registered worldwide primarily for the treatment of respiratory infections in food-producing animals. It has been argued that overuse or misuse of these ESCs has led to the emergence of a reservoir of ESC-resistant Enterobacteriaceae including *E. coli* and *Salmonella enterica* in livestock worldwide [4, 7, 8]. ESC resistance is predominantly plasmid-mediated and the most commonly identified ESC-resistance genes are *bla*_CMY-2_ and *bla*_CTX-M_ variants [9, 10]. Since the first report of ESC-resistant *E. coli* and *Salmonella* serovars in food-producing animals was linked to the use of ESCs in livestock, there has been increasing public health concern due to the direct transfer of resistant bacteria or the potential for the transfer of plasmid-mediated resistance to humans [2, 11–13]. In addition to the risk of acquiring ESC-resistant plasmids, prolonged carriage and persistence of ESC-resistance after the use of ESCs in livestock is an important complication to be considered [14].

Recent studies in humans and animals have shown that once ESC-resistance emerges in the host’s microbiota, it takes a considerable time for resistance genes distributed among autochthonous Enterobacteriaceae to be lost, even when direct antibiotic selection pressure is removed. This demonstrates the long-term impact of ESC-resistance on both human and animal health [15, 16]. A recent study in Denmark has shown that CTX-M- producing *E. coli* can persist for 1 year in the gut of pigs in the absence of direct selection pressure [16]. Studies in humans have shown that ESC-resistant *E. coli* can persist in the human gastrointestinal tract for up to 3–5 years [15, 17]. The reasons for this persistence are unclear, however co-selection through use of other antimicrobials, strain fitness and plasmid stability have been proposed as selection foci for prolonged carriage of ESC-resistant *E. coli* in livestock [15, 16, 18, 19]. A number of international studies have evaluated the carriage of ESC-resistant *E. coli* in livestock systems. However, little is known about the longitudinal carriage of ESC-resistance among commensal *E. coli* in pig herds and the maintenance of ESC-resistance plasmids in the absence of direct selection pressure for prolonged periods.

In this study, we describe the ecology and epidemiology of ESC-resistant *E. coli* in healthy pigs at a single piggery in Australia over a period of 4 years following the voluntary withdrawal of ESC from use on farm. Data collected have been used to comprehensively describe transmission, evolution and persistence of ESC-resistant plasmids within host commensal *E. coli* clones.

## Materials and Methods

### Sampling and antimicrobial use history

All work involving animals was undertaken as part of a veterinary investigation by the veterinarian in charge or was undertaken with the approval of the University of Adelaide Animal Ethics Committee (approval number S-2013-182). Rectal swabs were obtained from randomly selected pigs from an Australian piggery with a history of off-label ceftiofur use on an individual animal basis over the preceding 4 years. This investigation was performed by a specialist pig veterinarian as part of the herd health management plan for the piggery, and samples were submitted to the University of Adelaide Veterinary Diagnostic Laboratory for culture. While ceftiofur use occurred in the first year of the study (2013) as the primary control agent for post-farrowing *E.coli* scours in the farrowing area, it was discontinued in 2014 in response to the initial findings from this study. None of the animals sampled in years 2014, 2015 and 2016 had exposure to ceftiofur. However, from 2014 amoxicillin (Amoxil) at 500ppm was used in the creep feed for *Streptococcus suis* management. In addition, lincomycin was used as required in the grower feed for ileitis management and suppression of a minor colitis associated with *Brachyspira hyodysenteriae* and kitasamycin was used in the finisher feed for ileitis and *Erysipelas* infections.

Sampling of the pigs was opportunistic, however it was stratified by age and production class. A total of 490 rectal swabs were collected over four consecutive years from healthy pigs. This included rectal swabs from 2013 (*n* = 90), 2014 (*n* = 60), 2015 (*n* = 140) and 2016 (*n* = 200) (Table 1).

**Table 1:** Frequency of extended-spectrum cephalosporin-resistant *E. coli* carriage among different growing sheds in a pig farm from 2013–2016

Sampling location	Swabs (*n*)	ESC^R^ *E. coli* *n* (%)	95% CI
2013			
Farrowing shed	15	13 (86.6)	62.2–96.3
Weaner shed	35	32 (91.4)	77.6–97.0
Grower shed	20	16 (80)	58.4–91.9
Finisher shed	20	17 (85)	63.9–94.8
Total ESC^R^ *E. coli*	90	78 (86.6)	78.1–92.2
2014			
Farrowing shed	60	50 (83.3)	71.8–90.7
Total ESC^R^ *E. coli*	60	50 (83.3)	71.8–90.7
2015			
Farrowing shed	78	25 (32.1)	22.7–43.0
Growers shed	30	6 (20)	9.5–37.3
Finishers shed	32	0(0)	0.0–10.7
Total ESC^R^ *E. coli*	140	31(22.1)	16.1–29.7
2016			
Farrowing shed	80	14 (17.5)	10.7–27.3
Weaner shed	60	0(0)	0.0–6.0
Finisher shed	60	3(5)	1.7–13.7
Total ESC^R^ *E. coli*	200	17(8.5)	5.4–13.2

ESC^R^ - ESC-resistant

### Culture conditions and bacterial identification

Swabs were plated onto MacConkey agar (Thermofisher Scientific, Australia) supplemented with 4 µg/ml cefovecin (2013, Zoetis) or ceftiofur (2014–2016) (Sigma-Aldrich, Australia) and incubated overnight at 37 °C. Lactose fermenting colonies were identified, subcultured onto SBA (Thermofisher Scientific, Australia) and incubated at 37 °C overnight. *E. coli* was then identified from SBA plates by colony morphology, Gram stain and spot indole tests, and identifications were confirmed using MALDI-TOF (Bruker). One representative *E. coli* colony was subcultured on to SBA and preserved in brain heart infusion broth with 20% glycerol and stored frozen at −80 °C for later analysis. An additional *E. coli* isolate was selected from the selective agar if two morphologically distinct *E. coli* colony types were identified on the selective agar plate and on SBA.

### Antimicrobial susceptibility testing

Antimicrobial susceptibility was evaluated using minimum inhibitory concentration (MIC) testing by the broth-microdilution method as recommended by the Clinical Laboratory Standards Institute (CLSI) in 96 well plates [20]. Susceptibility to eight antimicrobials was assessed (ampicillin, ceftiofur, ceftriaxone, ciprofloxacin, florfenicol, gentamicin, tetracycline and trimethoprim/sulfamethoxazole). *E. coli* ATCC 25922 was used as a quality control strain as recommended. MIC results were interpreted based on clinical breakpoints according to CLSI VET01S (ampicillin, ceftiofur, florfenicol, gentamicin, tetracycline, trimethoprim/sulphamethoxazole) [21]. CLSI M100 was used where no breakpoints were available in the VET01S manual (ceftriaxone, ciprofloxacin) [22].

### DNA extraction for PCR assays

DNA extraction for preliminary screening was performed using 6% Chelex (Bio-Rad) as previously described with the exception that extractions were performed in 96 well plates [23].

### Molecular characterisation

The detection of the *bla*_CTX-M_ gene was performed using real-time PCR (RT-PCR) that amplifies all *bla*_CTX-M_ variants as previously described with minor variations [24]. Briefly, primers and probe CTX-A_fwd. (CGGGCRATGGCGCARAC), CTX-A_rev. (TGCRCCGGTSGTATTGCC) and CTX-A_probe (6FAM-CCARCGGGCGCAGYTGGTGAC-BHQ1) were combined at final working concentrations of 400 μM for each primer and 120 μM for the probe, with 2 μl of DNA extract and TaqPath qPCR Master Mix (Life Technologies). Reactions were run on a Quantstudio 6 Flex (Life Technologies) in fast mode, with samples showing a sigmoidal curve with a Ct ≤ 40 considered positive for the presence of the *bla*_CTX-M_ gene.

### Genome sequencing

Whole genome sequencing was performed on a subset of ESC-resistant *E. coli* isolates (*n* = 61). The isolates were selected on the basis of antimicrobial susceptibility results and year of isolation. DNA extraction of *E. coli* isolates for whole genome sequencing was performed using MagMAX-96 DNA multi-sample kit (Thermo Fisher Scientific). Genome sequencing was performed on Illumina MiSeq and NextSeq platforms using Nextera XT DNA library preparation and MiSeq V3 2 × 300 or NextSeq High Output v2 (300 cycles) kits according to the manufacturer’s instructions. Reads were de novo assembled using CLC Genomics Workbench v9.5.4, and contig files uploaded to the Centre for Genomic Epidemiology (CGE) (http://www.genomicepidemiology.org/) for screening for AMR genes, MLST and plasmid replicon type. Clermont Phylogenetic group was inferred from whole genome sequence data [25].

Core genome single nucleotide polymorphisms (SNPs) were extracted using the Nullarbor pipeline on 60 *E. coli* isolates [26]. Gubbins was further used to identify regions under horizontal transfer [27]. Core genome SNPS within these regions were removed and SNP data was imported into MEGA6 to generate a maximum parsimony tree [28]. One representative isolate (SA152) was also subject to PacBio Sequencing. The sequence data was de novo assembled using PacBio software and Quiver, and annotation was performed using RAST 28, with annotation editing performed using Geneious v10.1.3. The presence of genomic islands within plasmids was predicted using IslandViewer 4 [29].

Intimin subtypes (*eae*) were identified by BLASTn analysis against previously deposited *eae* sequences in GenBank. Isolates carrying intimin genes were analysed for the presence of bundle forming pilli (*bfp*) genes by mapping reads against the *E. coli* adherence factor plasmid pB171 (Genbank accession number: AB024946). In addition, all the sequenced isolates were screened for the carriage of porcine A/E-associated gene (*paa*) gene [30].

### Plasmid transfer by conjugation

Transferability of the resistance plasmid was performed by bacterial conjugation using *E. coli* J53Az^R^ recipient strain as previously reported using two randomly selected ESC-resistant *E. coli* isolates (SA210 and SA148) were used for this experiment [31]. The selection of the trans-conjugants was made on MacConkey Agar containing sodium azide (150 mg/L) and ceftiofur (4 µg/mL).

### Plasmid stability

Isolate SA152 was cultured onto sheep blood agar (SBA) (Edwards, Australia) and incubated overnight at 37 ^o^C. A single colony of SA152 was inoculated into 10 mL of Luria Bertani broth. After overnight incubation, 0.1 mL of each sample was inoculated into 9.9 mL of the corresponding broth or broth/antibiotic. This was repeated across 9 days. On the 9th day inoculums were plated in serial dilutions onto MacConkey agar (non-selective) and MacConkey agar (selective agar) with 4 mg/L calculate the CFU/mL. This was completed using a Tecan 1.5 robot with Scirobotics hardware.

## Results

### Detection of ESC-resistant *E. coli* carriage

Evaluation of ESC-resistant *E. coli* using selective agar demonstrated the relatively high carriage rates in 2013 (86.6%) and 2014 (83.3%); compared to 2015 (22%) and 2016 (8.5%) (Table 1). Samples obtained from finisher pigs in 2015 and 2016 were particularly noteworthy for the very low frequency of samples yielding ESC-resistant *E. coli* (0 and 5%, respectively) compared to 2013 (85 %) [Table 1].

Phenotypic characterisation of AMR by MIC determination confirmed on that all presumptive ESC-resistant *E. coli* isolated (*n* = 219) were resistant to ESCs (ceftiofur MICs ≥ 64 µg/mL). The majority of ceftiofur-resistant isolates were also resistant to ampicillin (100%), ceftriaxone (99.1%) and trimethoprim/sulfamethoxazole (99.1%). A smaller proportion of isolates were resistant to tetracycline (47.0%), gentamicin (16.4%) and florfenicol (11.4%). None of the isolates was resistant to fluoroquinolones (ciprofloxacin). All the isolates were positive for *bla*_CTX-M_ group 1 by RT-PCR, confirming the carriage of a *bla*_CTX-M_ gene.

### Molecular and genomic characterisation of ESC-resistant *E. coli* isolates

#### Phylogenetic characterisation of the isolates

A subset of representative isolates (*n* = 61) underwent whole genome sequencing to evaluate their full genomic characteristics. Genome sequencing confirmed the heterogeneous genetic make of the ESC-resistant *E. coli* isolates. Clermont phylogrouping revealed that the ESC-resistant *E. coli* isolates predominantly belonged to phylogenetic groups A (34.4%), C (29.5%) and B1 (24.6%) with remaining isolates classified as belonging to groups E (8.2%), D (1.6%) and B2 (1.6%). A total of 22 MLST types were identified among the ESC-resistant *E. coli* isolates, with ST10 (10/61), ST5440 (9/61), ST453 (6/61), ST2514 (6/61) and ST23 (4/61) being predominant. The CGE serotype predictor tool identified a number of serogroups including 18 O: types and 18 H: types (Table S1, supplemental material). The predominant serogroups identified among the ESC-resistant *E. coli* isolates were O8:H9 (7/61) and O8:H4 (7/61).

**Fig. 1:**
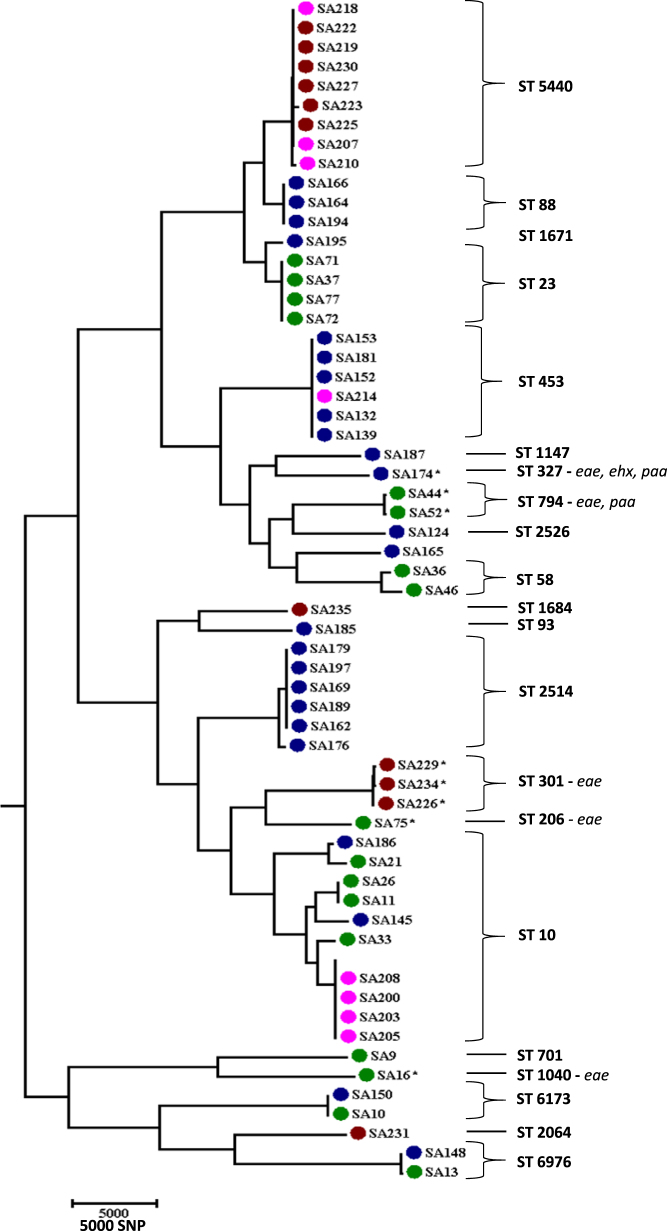
Dynamics and phylogenetic relationships of extended-spectrum cephalosporin resistant *E. coli* isolated from pigs over a period of four years from an Australian pig farm following the voluntary removal of ceftiofur. Transmission of IncI1-*bla*_CTXM-1_ plasmid (pCTXM1-MU2) in the pig farm among multiple *E. coli* lineages isolated from pigs of different age groups was demonstrated and a maximum parsimony tree using single nucleotide polymorphisms (SNPs) of extended-spectrum cephalosporin-resistant *E*. *coli* was generated. Year of isolation is marked with the colours green (2013), blue (2014), pink (2015) and brown (2016). MLST groupings for each cluster are also included. Isolates carrying the *eae*, *ehx* and *paa* gene are marked with an asterisk and written gene name

Phylogenetic characterisation using core genome SNPs also confirmed the polyclonal nature of the ESC-resistant *E. coli* population demonstrating a phylogenetically diverse ESC-resistant *E. coli* population in the piggery. The core-genome SNPs analysis mirrored the MLST analysis of the isolates (Fig. 1).

#### Antimicrobial resistance genes

**Fig. 2:**
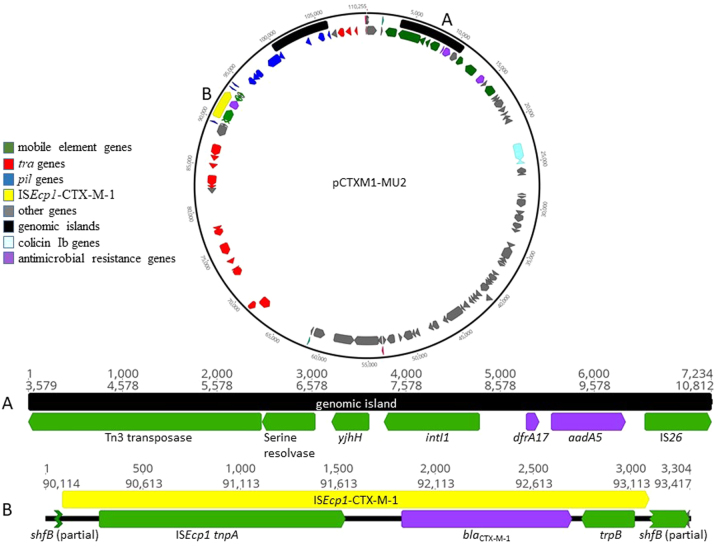
Genomic map of the plasmid pCTXM1-MU2 carried by extended-spectrum cephalosporin-resistant *E. coli* isolates belonging to multiple *E. coli* lineages in an Australian pig farm following the voluntary removal of ceftiofur. Positions of gene classes are indicated by colour. **a** Expanded view of the putative genomic island consisting of *aadA5* and *dfrA17* on a Tn3 transposase. **b** Expanded view of the IS*Ecp1*-CTX-M-1 region

**Fig. 3:**
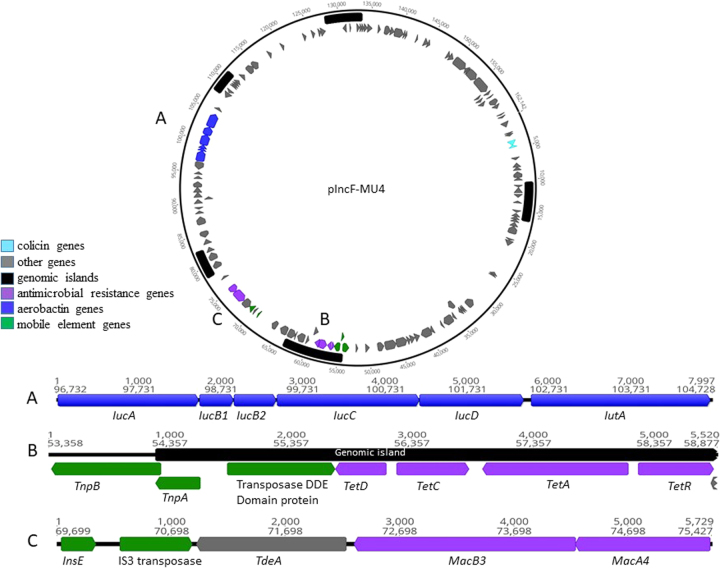
Genomic map of the plasmid pIncF-MU4 obtained from isolate SA152. Positions of gene classes are indicated by colour. **a** Expanded view of the aerobactin locus. **b** Expanded view of the tetracycline resistance cassette associated with an IS*2* transposase. **c** Expanded view of macrolide resistance genes associated with an IS*3* transposase

Genome sequencing revealed that the gene responsible for ESC resistance was *bla*_CTXM-1_, a commonly identified ESC resistance gene among livestock *E. coli*. Additional AMR genes (*aadA5, dfrA17* and *sul2)* were also identified among these isolates as detailed in Table S1, supplemental material, and as noted in Figs. 2 and 3. The *floR* resistance gene that encodes resistance to florfenicol (an important antimicrobial to treat various animal infections that is not used in human medicine) was present in three isolates.

### Plasmid characterisation

Preliminary plasmid characterisation revealed that an IncI1 plasmid was present among all the ESC-resistant *E. coli* isolates and contig mapping revealed that an IncI1 plasmid carried a *bla*_CTXM-1_ gene. *De novo* construction of the full plasmid was not possible with Illumina sequencing.

PacBio sequencing of one representative isolate (SA152) was used to detect and determine the full length sequence of an IncI1 ST-3 plasmid that carried *bla*_CTXM-1_. Read mapping of the rest of the isolates confirmed the presence of this plasmid, designated pCTXM1-MU2 (GenBank accession number MF152729) in all sequenced isolates.

The genetic map of pCTXM1-MU2 is shown in Fig. 2. The plasmid was 110,255 nucleotides in length, had a GC content of 51% and carried 23 IncI1 associated transfer genes. The *bla*_CTXM-1_ gene of interest was present in a region spanning nucleotide positions 91,939 – 92,814 and the resistance genes *dfr*A17, *aad*A5, and *sul*2 were present in the regions 8856–8987, 9118 – 9906 and 14,021–14,836, respectively. Upon BLASTn search, pCTXM1-MU2 displayed 95–100% query coverage and 99% identity to three previously reported IncI1 *E. coli* plasmids originating from chickens pC60-108, pC59-112 and pC49-108 (accession numbers KJ484635.1, KJ484637.1 and KJ484638.1 respectively), with MAUVE alignment of the genomes demonstrating the presence of largely conserved collinear coding blocks. All plasmids contained the *bla*_CTXM-1_ gene in association with insertion sequence IS*Ecp1*. In plasmid pCTXM1-MU2, IS*Ecp1* is flanked by 19 bp repeat sequences and is carried on a Tn*3* transposon.

In addition to the IncI1 plasmid, the ESC-resistant *E. coli* (SA152) also carried two other plasmids, an IncH12 plasmid 258,112 nucleotides in length designated pIncHI2-MU3 (GenBank accession number MF174859) and a smaller IncF plasmid of 162,142 nucleotides designated pIncF-MU4 (GenBank accession number MF174860) (Fig. 3). Plasmid pIncHI2-MU3 contained the AMR genes *bla*_TEM-12_ and *tetM* on Tn*3* transposons, along with *lnu(F)*, *aadA22*, *aac(3)-Iva* and *aph(4)-la*. The plasmid also carried a number of metal resistance genes including *copA*, *copB* and *pro*C copper resistance genes, *cusA/B/C/F* cation efflux genes and *silE* and *silP* silver resistance genes on a Tn*3* transposase. No other resistance genes or virulence genes of significance were present. Plasmid pIncF-MU4 carried Colicin Ia and Colicin E1 genes. AMR genes *tetA*, *tetC*, *tetD* and *tetR* were present on an IS*2* transposon, and the macrolide resistance genes *macA* and *macB* were present and associated with an IS*3* transposase element.

### Plasmid transferability and stability

The conjugation experiment revealed that the ESC-resistance-encoding pCTXM1-MU2 plasmid was easily transferred to *E. coli* J53. The efficiency of plasmid transfer from both donors (SA210 and SA148) were 3.4 × 10^−2^ and 2.7 × 10^−2^ respectively. Phenotypic antimicrobial susceptibility testing confirmed the resistance to ceftiofur and the transconjugates were positive for CTX-M by PCR.

Stability testing on SA152 in the absence of selection pressure with ESC revealed that the plasmid is stable within the host *E. coli*. No notable difference in CFU was observed after 9 overnight passages in LB broth plated on both MacConkey agar (1.9 × 10 ^11^ CFU/mL) and MacConkey agar infused with ceftriaxone (1.5 × 10 ^11^ CFU/mL), demonstrating the stability of the plasmid within the *E. coli* host without selection pressure.

To confirm the presence of pCTXM1-MU2 in isolates from multiple years, mapping of read files was performed against the curated plasmid sequence obtained from isolate SA152. Mapping of 12 isolates from 2013, 19 from 2014, 6 from 2015 and 8 from 2016 comprising 45 separate serotypes was performed. Coverage ranged from 97.7 to 100% (37 of 45 with 100% coverage) and pairwise alignment ranged from 95 to 98.5%. These results indicate the pCTXM1-MU2 plasmid was present in all isolates mapped, and that it was generally stable in genetic makeup over the four-year study period and across different *E. coli* serotypes.

### Virulence characterisation

The virulence screening revealed that none of the isolates could be classified as porcine enterotoxigenic *E. coli*, demonstrated by the lack of key virulence factors such as enterotoxins STa, STb and LT. The majority of the sequenced *E. coli* isolates appeared to be porcine commensal *E. coli* and lacked specific virulence factors that are usually identified among *E. coli* pathotypes including extra-intestinal pathogenic *E. coli* [32, 33]. However, a small (8/61) proportion of isolates were positive for the *eae* gene (intimin), but negative for the bundle forming pilus gene *bfpA* and the virulence regulator gene *perA*, a genotype that classifies the isolates as atypical enteropathogenc *E. coli* (aEPEC) [Table S1, supplemental material] [34, 35]. The intimin gene subtypes present in these isolates varied by serotype and year of isolation, with *eae*α1 present in three isolates from 2013 (two typed as ST794-O76:H7 and one as ST1040-O177:H45) and *eae*ε3 in three isolates from 2016 (three typed as ST301-O70:H2). The ST794-O76:H7 isolates also carried the porcine A/E-associated gene (*paa*) gene. A single isolate from 2013 carried *eae*-κ (typed as ST206-O49:H10). Another isolate from 2014 carried *eae*θ (typed as ST327-H8) and carried the enterohaemorrhagic *E. coli* (EHEC) hemolysin gene *ehx*A and *paa* gene. None of the aEPEC carried Shiga toxin genes, *bfp* genes or other fimbrial genes recognised as having a particular role in attachment to the intestinal epithelium.

The *eae* gene in *E. coli* is typically found on a locus of enterocyte effacement (LEE) pathogenicity island. When reads from all *eae* positive strains were mapped to a pathogenicity island reference sequence (GenBank accession AF022236), a consensus sequence contig with a query coverage of 98% and an identity of 99% was produced. While Illumina sequencing could not definitively assemble the LEE pathogenicity island to the chromosome, read mapping to AF022236 displayed coverage of 40 × over the 5′ and 3′ termini of this sequence, which corresponds to chromosomal regions, further providing evidence this is likely to be a chromosomally inserted island, as previously reported [36, 37].

## Discussion

In this study, we evaluated the carriage of ESC-resistant *E. coli* in a pig herd over a period of 4 years, and clarified the molecular characteristics of ESC-resistant *E. coli* and their associated plasmids responsible for ESC resistance. The major findings arising from this study are as follows: (i) ESC resistance identified among *E. coli* isolated in this study was attributed to the widespread dispersion and persistence of an IncI1 plasmid (pCTXM1-MU2) encoding the *bla*_CTXM-1_ gene. (ii) Genomic characterisation of ESC- resistant *E. coli* demonstrated that the ESC-resistant plasmid had moved into multiple commensal and several atypical enteropathogenic *E. coli* clonal lineages that harboured multiple LEE pathogenic island variants encoding the *eae* and/or *ehx* genes. (iii) Once ESC-resistant *E. coli* emerge in a livestock production system, it will take an extended period of time (at least 4 years) for significant reduction and/or elimination of ESC-resistant *E. coli* following withdrawal of the direct antimicrobial selection pressure. (iv) IncI1-*bla*_CTXM-1_ plasmids are globally disseminated, stable and highly transferable.

We have demonstrated that the dissemination of ESC-resistant *E. coli* and persistence of these clones in pig populations of different age categories is attributable to a highly transferable IncI1-*bla*_CTXM-1_ plasmid among different *E. coli* lineages. IncI1 plasmids carrying *bla*_CTXM-1_ are one of the most commonly identified plasmid-gene combinations imparting ESC resistance in pigs and poultry in Europe [38–40]. Genomic analysis of pCTXM1-MU2 demonstrated a close relationship with three IncI1-*bla*_CTXM-1_ plasmids isolated from poultry in Switzerland, with all *bla*_CTXM-1_ genes associated with an IS*ECP*1 insertion sequence which is commonly described [41]. The close genomic similarity of the plasmids, conservation of blocks of coding regions and common occurrence in livestock suggest that plasmids with an IncI1 backbone carrying the *bla*_CTXM-1_ gene in this configuration are relatively stable, prompting inquiry into this plasmid’s origin and its dissemination into an Australia pig herd.

The first possible hypothesis is that this plasmid was carried by a Gram-negative bacterium in livestock or their environment and was mobilised and transferred to commensal *E. coli* in the presence of antibiotic selection pressure from the use of ceftiofur as an off-label treatment for scours. Following this, continued administration of routinely used antimicrobials such as amoxicillin for the control of *Streptococcus suis* may have contributed to the persistence of the plasmid (pCTXM1-MU2) in pigs of older age groups and the piggery environment via co-selection.

In Australia, a ban on the live importation of pigs has been in place for over 30 years, just before ceftiofur was registered for use in livestock globally in the late 1980s and extending into the1990s. As a result, ESC-resistant plasmids may have been present in the gut of pigs before the use of ceftiofur, for example with the establishment of Australia’s pig herd, or alternatively, Australian environmental bacteria may harbour IncI1-*bla*_CTXM-1_ plasmids. This seems unlikely given that the plasmid is 99% similar to previously described IncI1 plasmids isolated from poultry *E. coli* strains in Europe.

The close relationship of the IncI1-*bla*_CTXM-1_ plasmid identified in this study to others identified in European livestock isolates, along with the potential stability of this plasmid-gene combination implies a more recent common source or pathway of introduction. This in turn leads to the second hypothesis that the plasmid may have been introduced via reverse zoonotic transmission from pig workers who have had recent contact with livestock or contaminated food products from countries where the IncI1-*bla*_CTXM-1_ plasmid is endemic. Human-to-animal transmission of resistant bacteria has been demonstrated by the emergence and spread of MRSA ST398 in Australian piggeries [42, 43]. A study using whole genome sequencing of MRSA ST398 isolates from Australian pigs suggests multiple incursions of European-origin clonal lineages, most likely from human carriers visiting European piggeries [43].

A third hypothesis is that the plasmid was introduced into the piggery environment via wild birds, with transfer of the plasmid occurring via interactions between endemic and migratory bird species. Our recent study has demonstrated that the detection of FQ and ESC-resistant *E. coli* clone ST744 in Australian pigs may have been introduced into Australian pigs via humans or migratory birds, since ST744 is commonly detected among humans and birds in Asian countries [44, 45].

In this piggery, the IncI1-*bla*_CTXM-1_ plasmid was detected in multiple *E. coli* lineages including potentially pathogenic atypical EPEC clones, demonstrating high transferability of this plasmid without apparent clonal discrimination. The *E. coli* clones identified in this study have previously been reported in humans and animals in other parts of the world. For instance, ST10 is a broad host range *E. coli* type that has been detected in pigs, poultry and humans. This study also shows that the IncI1-*bla*_CTXM-1_ plasmid has moved into extraintestinal pathogenic clonal lineages such as ST93 (O5:H4) an avian pathogenic *E. coli* (APEC) and ST453 (O23:H16), another clone known to cause extraintestinal disease in humans (urinary tract infections) and metritis in cattle. ST10 and ST453 containing CTX-M genes have previously been isolated from humans and animals, including pigs and seagulls [46–48].

A small number of the *E. coli* lineages that harboured IncI1-*bla*_CTXM-1_ also carried virulence factors associated with aEPEC. Atypical EPEC are categorised as *E. coli* that possess the LEE pathogenicity island and lack bundle-forming pili (BFP) and Shiga-toxin genes. A number of studies have associated aEPEC with paediatric diarrhoea, however, defining aEPEC as true pathogens is contentious due to the detection of these isolates in both diarrhoeic patients and control subjects [35, 49, 50]. In Australia, aEPEC have been isolated from clinical cases of diarrhoea in humans as and can be carried by healthy cattle and sheep [51]. In the present study, we identified eight aEPEC isolates belonging to different MLST groups and serotypes, confirming the presence of a heterogeneous group of ESC-resistant aEPEC strains in a single piggery. The carriage of intimin gene variants and *ehx* genes associated with EHEC pathotypes may indicate that these isolates are potential zoonotic pathogens. However, definitive testing in animal models would be required to confirm this as only a small number of studies have evaluated the full molecular characteristics, virulence potential and phylogeny of aEPEC [34, 35, 49, 50]. Further investigation is therefore warranted to fully characterise the virulence potential of these ESC-resistant aEPEC isolates and evaluate their ability to cause clinical disease in both humans and animals.

In this study, we have shown that once emerged, ESC-resistant *E. coli* is readily transmissible between pigs during different stages of production, resulting in an initial high carriage rate and persistence within the piggery over the 4-year study period. Genomic characterisation showed that the IncI1-*bla*_CTXM-1_ plasmid has remained highly stable over this period. However, despite continued detection of ESC-resistant *E. coli* over the course of the study, we demonstrated that withdrawal of ceftiofur had a significant impact on reducing ESC-resistant *E. coli* carriage in pigs in all age groups sampled, from 86.6% of pigs sampled in the first year following ceftiofur withdrawal, to 22.1 and 17.5% in the third and fourth years, respectively. This reduction was particularly apparent in finisher pigs, which may be related to the withholding period prior to slaughter when antimicrobial selection pressure is no longer a factor.

In conclusion, this study demonstrates the ecological dynamics of a highly transmissible, successful ESC-resistance plasmid in a pig herd and clarifies the genomic characteristics of ESC-resistant commensal *E. coli* harbouring the plasmid. This study shows that ceftiofur resistance may persist for a protracted period following removal of selection pressure, resulting in the emergence of multidrug resistance in both commensal *E. coli* and potentially virulent atypical EPEC isolates of significance to both human and animal health. This study also reveals that the IncI1-*bla*_CTXM-1_ plasmid pCTXM1-MU2 is a stable, highly transmissible plasmid that may confer additional fitness advantages to multiple *E. coli* lineages. Based on these results, a review of the current use of ESCs in livestock would be prudent, and ongoing surveillance combined with genomic characterisation of CIA-resistant bacteria is vital to mitigate the spread of antimicrobial resistance in livestock and its potential impact to human health.

## Electronic supplementary material

Table S1

Table S1 Legend
