# Supplementary material for: Dissemination and persistence of extended-spectrum cephalosporin-resistance encoding IncI1-blaCTXM-1 plasmid among Escherichia coli in pigs
Source: ISME J. 2018 Jun 13;12(10):2352–62. doi: 10.1038/s41396-018-0200-3 (PMC6155088; doi:10.1038/s41396-018-0200-3)
Supplement: Supplementary file 2 — Table S1 Legend [file 41396_2018_200_MOESM2_ESM.docx]

Table S1, supplemental material. Metadata of 61 extended-spectrum cephalosporin-resistant *E. coli* subjected to whole genome sequencing.
